# Symmetry and range limits in importance indices

**DOI:** 10.1002/ece3.1649

**Published:** 2015-09-26

**Authors:** Tal Seifan, Merav Seifan

**Affiliations:** ^1^ P.O.B 3808499000Midreshet Ben GurionIsrael; ^2^ Mitrani Department of Desert EcologySwiss Institute for Dryland Environmental and Energy ResearchJacob Blaustein Institutes for Desert ResearchBen‐Gurion University of the Negev8499000Midreshet Ben‐GurionIsrael

**Keywords:** Cimp, competition, facilitation, Iimp, importance, intensity, neighbor effect

## Abstract

Recently, Mingo has analyzed the properties of *I*_imp_, an importance index, and demonstrated that its range is not symmetrical. While agreeing with this comment, we believe that more light needs to be shed on the issue of symmetry in relation to such indices. Importance indices are calculated using three values: performance of the organism in the absence and in the presence of neighbors and maximum performance of the organism in ideal conditions. Because of this structure, importance indices can hardly ever achieve symmetry along the whole range of potential performances. We discuss the limitation of the symmetry range for different symmetry types and for both additive and multiplicative indices. We conclude that importance indices, as other interactions indices, are practical tools for interpreting ecological outcomes, especially while comparing between studies. Nevertheless, the current structure of importance indices prevents symmetry along their whole range. While the lack of “perfect” symmetry may call for the development of more sophisticated importance metrics, the current indices are still helpful for the understanding of biological systems and should not be discarded before better alternatives are well established. To prevent potential confusion, we suggest that ecologists present the relevant index symmetry range in addition to their results, thus minimizing the probability of misinterpretation.

## Introduction

Interactions among individuals are one of the fundamental ecological forces that shape dynamics in space and time at all organization levels. Due to the significant role of interactions, and to the increasing interest in comparing results between studies, researchers strive to use metrics that can bridge between different units and scales. A common and practical approach is to transform the interactions into indices (Weigelt and Jolliffe 2003). When properly applied, such indices supply information about the strength and direction of interactions, independent of the units of the study, simplifying between‐study comparisons. Unfortunately, the usage of indices has disadvantages, including statistical and mathematical problems (e.g., Oksanen et al. 2006), disagreements regarding the correct measurements to apply (Freckleton et al. 2009), and theoretical problems with their interpretation (Rees and Freckleton 2012). Nevertheless, indices of interactions are a popular tool because they are considered simple and practical to use due to their standardized range.

Interaction indices deal with either the intensity or the importance of interactions (Welden and Slauson 1986; Brooker et al. 2005). The distinction stems from the relative role of the interactions: while intensity refers to the absolute impact of neighbors on the performance of the target organism, importance estimates neighbor effects relative to the effects of the environment (Welden and Slauson 1986). The interest in importance indices was lately intensified with discussions on several unresolved issues regarding today's metrics (Freckleton et al. 2009; Kikvidze and Brooker 2010; Freckleton and Rees 2011; Kikvidze et al. 2011a,b; Rees and Freckleton 2012; Brooker et al. 2013). In 2010 Seifan et al. have pointed out that the most popular index for measuring importance, *C*_imp_ (Brooker et al. 2005), is problematic when positive interactions occur. We followed our critique by suggesting a simple adjustment to the index, which we named *I*_imp_ (Seifan et al. 2010). *I*_imp_ is calculated using the contribution of biotic (*N*_imp_ – interactions with neighbors) and abiotic (*E*_imp_ – local environmental effects) factors to the organism performance. *N*_imp_ is defined as the difference between the performance of the organism with and without neighbors (*P*_+N_ and *P*_−N_, respectively):

$$N_{\mathrm{imp}}=P_{+N}-P_{-N},\text{where}-\_-N\leq N_{\mathrm{imp}}<\infty$$

*E*_imp_ is derived from the difference between the maximum organisms performance (*MP*_±N_) and its performance in the specific site in the absence of neighbors (*P*_−N_):

$$E_{\mathrm{imp}}=P_{-N}-MP_{\pm N},\text{where}-MP_{\pm N}\leq E_{\mathrm{imp}}<\infty$$

The importance of neighbor interactions is defines as:

$$\left\{\begin{array}{c} I_{\mathrm{imp}}=\frac{N_{\mathrm{imp}}}{\left|N_{\mathrm{imp}}\right|+\left|E_{\mathrm{imp}}\right|}\left|N_{\mathrm{imp}}\right|+\left|E_{\mathrm{imp}}\right|>0\\ 0\left|N_{\mathrm{imp}}\right|+\left|E_{\mathrm{imp}}\right|=0 \end{array}\right.$$

Recently, Mingo (2014b) correctly pointed out that the range of *I*_imp_ is [−1, 0.5] and not [−1, 1] as we erroneously claimed. Following his comment, and in relation to Mingo's further discussion on the topic (Mingo 2014a,b), we realized that there is a need to shed light on one of the required properties of interaction metrics overall, and especially importance indices – index symmetry.

Armas et al. (2004) listed the preferable properties for interaction indices. At the top of the list of ideal properties was the request for symmetry (Armas et al. 2004). For example, for an index with symmetry around zero, negative and positive interactions of the same absolute size will result with the same index value, but with different signs. The main advantage of symmetrical indices stems from their intuitiveness. An asymmetric index, even if it pivots around a well‐defined centre (e.g., around zero), is more difficult to comprehend than a symmetric index. Although symmetry is not a mathematical requirement, the lack of symmetry in ecological metrics was criticized because it increases the probability of biased interpretations, particularly when comparing results between different studies (see also Hedges et al. 1999; Oksanen et al. 2006). While achieving symmetry in intensity indices is relatively easy (Armas et al. 2004; Seifan et al. 2010), the situation is more complicated when evaluating importance of interactions. For example, *C*_imp_ is nonlinear in case of competition and linear in case of facilitations, and *I*_imp_ is symmetrical only within a specific range (for calculation of the symmetrical range of *I*_imp_, see in the discussion below). The complexity arises because the goal of importance indices was to estimate how much (if at all) an organism performance is affected by neighbors, relative to its performance under ideal conditions, resulting with metrics with two reference points simultaneously.

## Potential types of symmetry in indices

In the following, we will first discuss the structure of importance indices and the meaning of symmetry in this context. We will then analyze the range of symmetry and show that symmetry cannot be achieved along the whole range of such metrics due to their nature.

Importance indices are calculated using three values: performance of the organism in the absence of neighbors (*P*_−N_), performance of the organism in the presence of neighbors (*P*_+N_), and maximum performance of the organism, regardless of the presence/absence of neighbors (*MP*_±N_), often defined as the maximum value measured in the specific experiment (e.g., Ariza and Tielbörger 2011; Armas et al. 2011; Soliveres et al. 2011; Bennett and Cahill 2012; Howard et al. 2012; Le Bagousse‐Pinguet et al. 2014). Note that although being a highly popular choice, there are alternative methods to assess maximum performance (see discussion in Seifan et al. 2010; Mingo 2014a).

When discussing symmetry features of indices based on these values, *P*_−N_ is regarded as the pivot point. Hence, when *P*_+N_ = *P*_−N_, the value of such indices is zero, regardless of the maximum performance of the organism. Any other value of (*P*_+N_) will produce a nonzero index value. For the sake of convenience, we divide the explanation into two types of symmetry. We will first demonstrate our arguments using indices based on additive scales (as *C*_imp_) and then discuss multiplicative indices.

Definition of an additive symmetrical index of importance: For the interaction effect *X*,

$$\left\{\begin{array}{c} -P_{-N}\leq X<0\text{Competition}\\ X=0\text{Interactions do not affect the target plant}\\ 0<X<\infty \text{Facilitation} \end{array}\right.$$

a theoretical additive index *A* will be symmetrical if and only if for every *X*,

$$A_{\left(P_{-N}+X\right)}=-A_{\left(P_{-N}-X\right)}$$

### Type I symmetry – zero as the reference point

**Figure 1:**
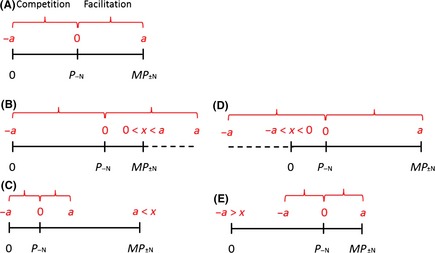
Symmetry limitations in additive indices. Continuous black lines represent actual range of performance values. Dashed lines represent values outside the possible performance range. Red – symmetry range of a theoretical importance index; the values (in red) are for demonstration purposes only: *a* – the index value at the positive edge of the symmetrical range; *x* – the index value at points of interest (*P*_+N_ = 0 or *P*_+N_ = *MP*_±N_). (A) symmetry range matches performance range of the organism; Symmetry range in symmetry type I when (B) *P*_−N_ > 1/2 *MP*_±N_; and (C) *P*_−N_ < 1/2 *MP*_±N_; Symmetry range in symmetry type II when (D) *P*_−N_ < 1/2 *MP*_±N_; and (E) *P*_−N_ > 1/2 *MP*_±N_.

In this case, the minimum possible performance value (*P*_+N_ = 0; i.e., competitive exclusion) will always be the lowest value of the symmetry range. For such additive indices, the symmetry range is 0 ≤ *P*_+N_ ≤ 2*P*_−N_, and only when *P*_−N_ = 1/2 *MP*_±N_ will the index be symmetrical for the whole range (Fig. 1A). If the performance of the organism without neighbors is larger than half the maximum performance (*P*_−N_ > 1/2 *MP*_±N_), the theoretical range of symmetry will exceed the maximum performance value. Under such conditions, the actual index values in case of a positive effect of neighbors will always be smaller than the potential theoretical values defined by the index symmetry (Fig. 1B). On the other hand, when the performance of the organism without neighbors is smaller than half the maximum performance (*P*_−N_ < 1/2 *MP*_±N_), the symmetry range is smaller than the maximum performance of the organism. Therefore, some actual measured values are bound to exceed the symmetry area of the index when neighbors have positive effect on the target organism (Fig. 1C).

### Type II symmetry – *MP*_±N_ as the reference point

In this case, the maximum possible performance value (*P*_+N_ = *MP*_±N_; i.e., when the best performance of the target organism is achieved under a facilitation scenario) will always be the highest value of the symmetry range. The symmetry range under type II symmetry is (2*P*_−N_ − *MP*_±N_) ≤ *P*_+N_ ≤ *MP*_±N_, and as in type I symmetry, a completely symmetrical index can be generated only when *P*_−N_ = 1/2 *MP*_±N_ (Fig. 1A). When *P*_−N_ < 1/2 *MP*_±N_ the symmetrical range goes beyond the minimum value of the index, so that even when neighbors cause extinction of the target individual (*P*_+N_ = 0), the calculated value of the index will be larger than the theoretical minimum (Fig. 1D). When *P*_−N_ > 1/2 *MP*_±N_, the symmetry range is smaller than the index range, hence values close to competitive exclusion of the target organism (*P*_+N_ = 0) are excluded from the symmetry range (Fig. 1E). Note that *I*_imp_, has a type II symmetry: while the value at competitive exclusion (*P*_+N_ = 0) changes with measurements of the specific study

$$\left(I_{\mathrm{imp}\left(P_{+N}=0\right)}=\frac{P_{+N}-P_{-N}}{MP_{\pm N}-P_{+N}}=\frac{0-P_{-N}}{MP_{\pm N}-0}=-\frac{P_{-N}}{MP_{\pm N}}\right)$$

the value at maximum possible performance (*P*_+N_ = *MP*_±N_) is constant, regardless of the specific measurements

$$\begin{array}{c} (I_{\mathrm{imp}\left(P_{+N}\;=\;MP_{\pm N}\right)}=\frac{P_{+N}-P_{-N}}{MP_{\pm N}-2P_{-N}+P_{+N}}\\ \;=\frac{MP_{\pm N}-P_{-N}}{MP_{\pm N}-2P_{-N}+MP_{\pm N}}=\frac{1}{2}) \end{array}$$

Overall, it is clear that regardless of the approach to symmetry, an additive importance index cannot be fully symmetrical along the performance range. In order to achieve index symmetry in the whole performance range [0, *MP*_±N_] a type I symmetry should be used when $P_{-N}\geq \frac{MP_{\pm N}}{2}$, while a type II symmetry should be used when $P_{-N}\leq \frac{MP_{\pm N}}{2}$. Nevertheless, although this solution will result with symmetry for the whole performance range of the organism, some values will have a purely theoretical symmetrical equivalent, that cannot result from actual measurements.

### The case of multiplicative symmetry

A multiplicative approach is found, for example, in the intensity index suggested by Armas et al. 2004.

Definition of a multiplicative symmetrical index of importance: For the interaction effect *α*,

$$\left\{\begin{array}{c} 0\leq \alpha <1\text{Competition}\\ \alpha =1\text{Interactions do not affect the target plant}\\ 1<\alpha <\infty \text{Facilitation} \end{array}\right.$$

a theoretical multiplicative index *M* will be symmetrical if and only if for every *α*,

$$M_{\left(\alpha \times P_{-N}\right)}=-M_{\left(\frac{1}{\alpha }\times P_{-N}\right)}$$

**Figure 2:**
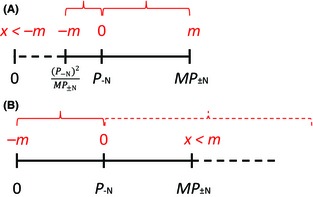
Symmetry limitations in multiplicative indices. Continuous black lines represent actual range of performance values. Dashed lines represent values outside the possible performance range. Red – symmetry range of a theoretical importance index; the values (in red) are for demonstration purposes only: *m* – index value at the positive edge of the symmetrical range; *x* – index value at points of interest (*P*_+N_ = 0 or *P*_+N_ = *MP*_±N_). (A) symmetry within the individual performance range; (B) symmetry outside the individual performance range.

A multiplicative symmetrical index, as defined above, inflicts even more complex problems on the potential development of importance indices. By definition, the organism's best performance is *MP*_±N_. Let us define $\alpha^{\prime }=\frac{MP_{\pm N}}{P_{-N}}$. Multiplicative importance indices can be symmetrical in the range $\left[\frac{P_{-N}}{\alpha^{\prime }},\alpha^{\prime }\times P_{-N}\right]$, which is equivalent to $\left[\frac{{\left(P_{-N}\right)}^{2}}{MP_{\pm N}},MP_{\pm N}\right]$ and will always be within the organism's performance range [0, *MP*_±N_]. Hence, *MP*_±N_ will always be within the symmetry range (Fig. 2A). However, when considering complete exclusion (*P*_+N_ = 0), the index properties become problematic: for *α*_1_ = 0, a symmetrical index that will include complete exclusion must be in the range $\left[\alpha_{1}\times P_{-N},\frac{P_{-N}}{\alpha_{1}}\right]$, which is equivalent to [0, ∞). Such an index would be too problematic to use because it ignores *MP*_±N_ (Fig. 2B). Moreover, such an index will be over‐sensitive close to complete exclusion (*P*_+N_ ≈ 0), causing small changes in performance, which may not have a significant biological meaning (and may even be within measure error) to generate index values that are different in orders of magnitude.

## Discussion

**Figure 3:**
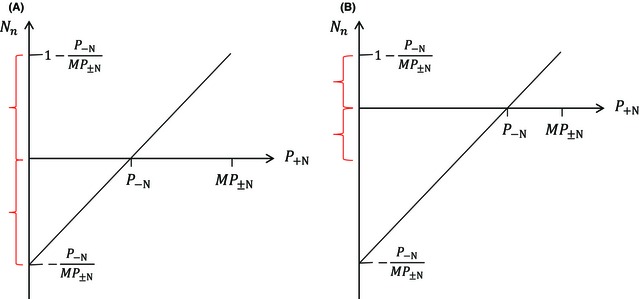
Mingo's “Normalized neighbor effect,” using the adjustments mentioned in the text. (A) The index symmetry range (red) matches the performance range of the organism ($P_{-N}=\frac{MP_{\pm N}}{2}$). (B) Symmetry range (red) when *P*_−N_ > 1/2 *MP*_±N_.

Interaction importance refers to the effect of specific interactions relative to the impact of the environment (which includes both biotic and abiotic factors) on the performance of the organism (Seifan et al. 2010; Brooker et al. 2013). While a highly convenient tool for the interpretation of such interactions, metrics that attempt to transform this notion to a comparable value encountered difficulties maintaining practicality as listed by Armas et al. (2004). For example, Mingo (2014a) suggested a new approach to the assessment of neighbors versus environmental effects on performance that he named “Normalized neighbor effect” (*N*_n_; for uniformity reasons the following adjustments were conducted: (1) the index was set to have positive values for facilitation and negative values for competition; (2) the index arguments are presented using the above nomenclature, rather than the one found in Mingo 2014a). The index standardizes the difference in performance in the absence and presence of neighbors by scaling it relative to the maximum performance of the organism (*MP*_±N_), using the equation $N_{n}=\frac{P_{+N}-P_{-N}}{MP_{\pm N}}$. However, even if one accepts the arguments for using this index as a compromise between intensity and importance indices (see Mingo 2014a), the index suffers from the asymmetry problems we demonstrated above (Fig. 3). *N*_n_ is symmetrical around *P*_−N_ (*N*_n_ = 0, meaning that interactions had no effect over the observed target organism – equivalent to the abovementioned symmetry types) with the range $\left(-\frac{P_{-N}}{MP_{\pm N}}\leq N_{n}\leq 1-\frac{P_{-N}}{MP_{\pm N}}\right)$. As before, this index is symmetrical over the whole range if and only if $P_{-N}=\frac{MP_{\pm N}}{2}$ (Fig. 3A).

As far as we can perceive, the only mathematical solution to satisfy the requirement for symmetry at the whole range of the organism performance [0, *MP*_±N_] is to integrate the concept of importance index when facilitation occurs (in the range [*P*_−N_, *MP*_±N_]) with the concept of intensity index when competition occurs (in the range [0, *P*_−N_]). Obviously such a mathematical solution has no ecological meaning and therefore should not be used.

We conclude that importance indices cannot satisfy the practical requirement of symmetry between positive and negative interactions. While this conclusion may be disappointing, we firmly believe that importance indices are an informative tool that should be further used, albeit with caution. In particular, we suggest that ecologists add to the output presentation a calculation of the symmetrical range of the index, as reflected by their specific dataset. Such an addition will help preventing a biased interpretation of the outcome, while still maintaining the practical aspects of the usage of indices. In order to do that, one should determine the absolute value of the symmetrical area's edges $\left(\text{Edge}=\text{min}\left(\frac{P_{-N}}{MP_{\pm N}},\frac{1}{2}\right)\right)$ and set the symmetrical range accordingly (Symmetrical range = [−Edge, Edge]).

For many purposes, importance indices are a very useful tool that should not be discarded, even if some of the criticism they received is justified (e.g. Rees and Freckleton 2012). Indeed, there is room for the development of new methods that will better use the information latent in the abovementioned measurements while maintaining the theoretical ideas behind the notions of importance (e.g. Wilson 2007; Rees and Freckleton 2012). However, the development of such methods should be conducted carefully, so the intuitive and straightforward features of today's metrics, that make them so popular among ecologists, will not be lost.

## Conflict of Interest

None declared.
